# Effect of Sprouting, Fermentation and Cooking on Antioxidant Content and Total Antioxidant Activity in Quinoa and Amaranth

**DOI:** 10.3390/ijms252010972

**Published:** 2024-10-12

**Authors:** Martina Vento, Clara Maria Della Croce, Lorenza Bellani, Eliana Lanfranca Tassi, Maria Cristina Echeverria, Lucia Giorgetti

**Affiliations:** 1 Institute of Biology and Agricultural Biotechnology (IBBA), National Research Council, 56124 Pisa, Italy; martina.vento@ibba.cnr.it (M.V.); claramaria.dellacroce@cnr.it (C.M.D.C.); 2 Department of Life Sciences, University of Siena, 53100 Siena, Italy; 3 Research Institute on Terrestrial Ecosystems (IRET), National Research Council, 56124 Pisa, Italy; elianalanfranca.tassi@cnr.it; 4 eCIER Research Group, Department of Biotechnology, Universidad Técnica del Norte, Av. 17 de Julio 5–21 y Gral. José María Córdova, Ibarra 100150, Ecuador; mecheverria@utn.edu.ec

**Keywords:** *Chenopodium quinoa*, *Amaranthus caudatus*, antioxidant activity, polyphenols, flavonoids, mineral nutrient content, germination, fermentation, antimutagenic activity, functional food

## Abstract

The study of different processing techniques, such as sprouting, cooking and fermentation, can help to develop new products for human health. In this work, raw, cooked and fermented seeds and germinated seeds of *Chenopodium quinoa* Willd. var. Tunkahuan and *Amaranthus caudatus* L. var. Alegrìa were compared for the content of antioxidant molecules, total antioxidant capacity and mineral elements. Fermentation was induced spontaneously, with the yeast *Saccharomyces cerevisiae*, with the bacterium *Lactobacillus plantarum* and with both microorganisms, for 24 and 48 h. The increase in antioxidant molecules and antioxidant activity was induced by germination, by 24 h of spontaneous fermentation (polyphenols and flavonoids) and by 24 h of *L. plantarum* fermentation (total antioxidant activity) for both species. Germinated seeds of the two plants showed higher values in respect to seeds of macroelements and microelements. No genotoxic but rather protective effects were determined for seed and germinated seed extracts using the D7 strain of *S. cerevisiae*, a good tool for the evaluation of protection from oxidative damage induced by radical oxygen species (ROS) in cells and tissues. Therefore, the two varieties could be very suitable for their use in human diet and in supplements, especially as germinated seeds or as fermented foods.

## 1. Introduction

Quinoa (*Chenopodium quinoa* Willd.) and amaranth (*Amaranthus caudatus* L.) are pseudocereals of Andean origin belonging to the Chenopodiaceae and Amarantaceae families, respectively [1,2]. Quinoa and amaranth have been particularly valorized in recent decades for their high nutritional value [3,4,5], becoming important for global marketing. Known as “golden grains” and “food of the past for people of the future,” research by the National Academy of Sciences (NAS) has ranked quinoa and amaranth as the best plant food for humans [6]. Quinoa and amaranth present economic profitability for the development of products in the pharmaceutical, cosmetic and food sectors [7]. The entire plant can be used as green fodder for livestock: positive results have in fact been highlighted on the production performance and health of animals such as cattle, horses, pigs, sheep and poultry [8,9]. For human consumption, mainly quinoa and amaranth seeds and germinated seeds are used which can be consumed either fresh, sautéed or cooked, being tasty and with high nutritional qualities [10]. Both species have high content of macro- and micronutrients, amino acids, proteins, phenolic acids, vitamins, flavonoids and fatty acids, so their potential use in functional foods and nutraceuticals has been evaluated [11]. In fact, the inclusion of pseudocereals in the diet would be useful in maintaining human health and well-being, helping to prevent various pathologies such as neoplasms, diabetes and cardiovascular and degenerative diseases [12].

Natural antioxidants contained in new natural drugs and functional foods have recently received attention for their ability to counteract the deleterious effects of an excess of free radicals and the pathologies associated with them [13]. Specifically, the antioxidant properties of quinoa and amaranth can be justified by the presence of compounds such as phytosterols, polyphenols, vitamins, squalene and bioactive peptides [14,15]. Phenolic compounds include phenolic acids, flavonoids and tannins responsible for a wide range of physiological effects on the plant, especially defense-related mechanisms against ultraviolet radiation, pathogens and herbivores [16]. When introduced in the diet, polyphenols have anti-inflammatory, antimicrobial, cardiovascular protective and anti-carcinogenic activities [17] as they act as electron donors and acceptors, neutralizing peroxides and hydroperoxides and activating and deactivating metals [18]. In quinoa and amaranth, phenolic compounds such as gallic, vanillic, ferulic acid and their derivatives and flavonoids such as kaempferol, quercetin, rutin and their glycosides and tannins have been detected, mostly with antioxidant, anti-inflammatory, anti-diabetic and anticancer properties [19,20,21,22,23]. The abovementioned compounds could be important in developing functional foods to improve human health [24]. In fact, quinoa and amaranth are commercially added to cereals as flours and flakes or incorporated into salads, breakfast dishes, soups and sauces. Their flours can be used in the preparation of pasta, biscuits, bread, pies, puddings, crackers, pancakes and muffins, totally or partially replacing wheat flour [25]. Unlike wheat, amaranth and quinoa are gluten-free and therefore accessible to celiac consumers [26] and used as supplements due to their high protein content [7]. Furthermore, their use can also be encouraged, due to the high content of calcium and manganese, to promote bone health and regulate blood sugar levels [27], especially for people with diabetes in adulthood [28].

The organoleptic and nutritional characteristics of amaranth and quinoa can be enhanced through different processing techniques, including germination, cooking and fermentation. Although pseudocereals are mostly consumed after cooking, thermal treatments can lead to significant loss of soluble nutrients, in particular minerals, influencing their physico-chemical properties depending on the element considered and the type of plant variety [29].

Germination can improve the nutritional quality of a seed by eliminating or inactivating some anti-nutritional factors, increasing the digestibility of proteins and starch and causing changes in the distribution of secondary metabolites [30]. In many studies conducted on quinoa and amaranth, germination led to the decrease in lipids and phytic acid, while there was an increase in the content of fiber, vitamins, mineral salts and proteins, with an improvement in digestibility compared to seeds [31]. Moreover, an increase in phenolic compounds, anthocyanins, flavonoids and antioxidant activity was determined during germination, when compared to non-germinated seeds [14,32,33,34,35,36]. 

Fermentation is another food processing technology based on the growth and metabolic activity of microorganisms (e.g., bacteria, yeasts and filamentous fungi and their enzymes) used to stabilize and improve the nutritional quality of matrices. Fermentation can occur by microorganisms naturally present in the substrate or the processing environment (spontaneously fermented foods) or by microorganisms added as an initial culture (culture-dependent fermented foods). In both cases, fermentation occurs if a suitable substrate, one or more appropriate microorganisms, and adequate environmental conditions, such as temperature, pH and moisture content, coexist [37]. During the fermentation process, numerous biochemical changes occur in the food matrix, and the nutritional and anti-nutritional components are modified in terms of bioactivity and digestibility. For example, in unfermented cereals, phenolic compounds are mainly present in bound form, i.e., conjugated with sugars, fatty acids or proteins, while during fermentation, these are bioconverted into free forms for enzymatic activity or metabolic activity of fermenting microorganisms [38]. Free form of phenolic compounds has increased bioaccessibility and the release of free aglycones, polyphenols with a potential increase in antioxidant activity [39]. On the other hand, during some types of fermentation, a decrease in the content of free phenolic compounds can occur due to bonds with other molecules present in the food matrix or due to degradation by microbial enzymes with hydrolysis carried out by specific microbial strains [40].

In this work, seeds and germinated seeds of quinoa var. Tunkahuan and amaranth var. Alegria, both cultivated in the same area of Ecuador, were compared for the first time for the content of antioxidant substances, total antioxidant capacity and mineral nutrients, analyzing raw, cooked and fermented products. Furthermore, the potential genotoxic and/or protective effect of the extracts of the two plant species was evaluated using the D7 yeast strain *Saccharomyces cerevisiae*, a model system for the evaluation of genotoxicity and protection against oxidative damage induced by ROS.

## 2. Results

### 2.1. Total Polyphenol and Flavonoid Content and Total Antioxidant Capacity in Raw Seeds, Cooked Seeds and Germinated Seeds

**Figure 1:**
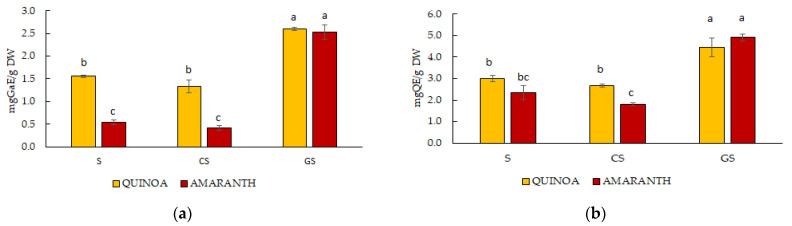

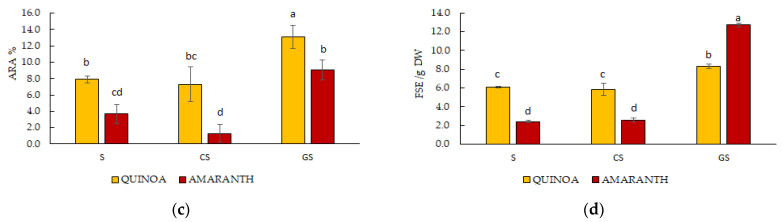
Spectrophotometric analysis of total polyphenol content (**a**), total flavonoid content (**b**), antioxidant capacity by DPPH (**c**) and FRAP (**d**) assays of raw seeds (S), cooked seeds (CS) and germinated seeds (GS) of quinoa and amaranth. Values were expressed as mg of gallic acid equivalent/gram of dry weight (mg GaE/g DW) for polyphenols, as mg of quercetin equivalent/gram of dry weight (mg QE/g DW) for flavonoids, as % of Anti Radical Activity (ARA%) for DPPH assay and as mg FS (ferrous sulphate) equivalents/gram of dry weight (mg FSE/g DW) for FRAP assay. Values were the average of three determinations ± SD; values followed by different letters were statistically significant according to Tukey’s test (*p* ≤ 0.05).

As to the content of polyphenols (Figure 1), the amount in raw and cooked seeds of quinoa ranged around 1.5 mg GaE/g DW, while in raw and cooked seeds of amaranth it was significantly lower, around 0.5 mg GaE/g DW. No effect was induced by cooking, while sprouting significantly increased the polyphenol content, which reached about 2.5 mg GaE/g DW in both species (Figure 1a). As to flavonoid content, no significant differences were observed in quinoa between raw and cooked seeds, with values around 3 mg QE/g DW. In amaranth, the content was similar in raw seeds but slightly lowered after cooking to 1.8 mg QE/g DW. On the other hand, sprouting significantly increased flavonoid content to a value of around 4.5–5.0 mg QE/g DW in both species (Figure 1b).

The antioxidant activity, evidenced by DPPH (Figure 1c) and FRAP (Figure 1d), indicated a similar trend in raw and cooked seeds of quinoa and amaranth, the latter being significantly lower than the former. Sprouting increased the antioxidant activity in both species, ARA being significantly higher in quinoa than in amaranth (13.07% and 9.06%, respectively) and FRAP in amaranth (12.5 mg FSE/g DW) than in quinoa (8.5 mg FSE/g DW) (Figure 1c). From these results, it was observed that the antioxidant activity of the germinated seeds of both species was significantly higher than that of the seeds, with quinoa having the highest ARA and amaranth the highest FRAP.

### 2.2. Fermentation

After 24 h of fermentation without sterilization (Table 1) in the different experimental conditions (spontaneous fermentation, fermentation with *S. cerevisiae*, with *L. plantarum*, with *S. cerevisiae* + *L. plantarum*), a general significant increase in the content of polyphenols was observed both in quinoa and in amaranth, either in seeds or germinated seeds.

In particular, the amount of polyphenols in unfermented seeds of quinoa was 1.5 mg GaE/g DW, and the highest content was reached after fermentation with *S. cerevisiae* + *L. plantarum* (10.54 mg GaE/g DW, 7 times greater than the unfermented seeds). In the germinated seeds of non-fermented quinoa, 2.6 mg GaE/g DW of polyphenols was found, and the highest content was after spontaneous fermentation (11.78 mg GaE/g DW), being 4.5 times greater than the unfermented material. In amaranth, a similar trend was observed, with the best increase in total polyphenols found after spontaneous fermentation from 0.54 to 5.59 mg GaE/g DW (10 times more) in seeds and from 2.54 to 9.92 mg GaE/g DW (about 4 times higher) in germinated seeds. 

Concerning flavonoids, all the conditions of fermentation induced an increase except for a decrease after fermentation with *L. plantarum* in quinoa seeds (from 3.00 to 1.68 mg QE/g DW) and with *S. cerevisiae* and *S. cerevisiae + L. plantarum* in amaranth seeds (from 2.35 to 1.58 and 1.31 mg QE/g DW, respectively). In quinoa seeds, the highest values were obtained after fermentation with *S. cerevisiae* + *L. plantarum* (4.87 mg QE/g DW) (1.6 times more in respect to unfermented seeds) and in amaranth after spontaneous fermentation (6.1 mg QE/g DW) (about 2.6 times more than unfermented seeds). In the germinated seeds of both species, the highest flavonoid content was detected after spontaneous fermentation (12.81 in quinoa and 9.41 mg QE/g DW in amaranth, 2.9 and 1.9 times more than unfermented germinated seeds, respectively) and a slight decrease only after fermentation with *S. cerevisiae* in amaranth.

The antioxidant activity, determined by ARA% and FRAP, was generally increased by fermentation. In quinoa, the highest values after DPPH assay were found in seeds after fermentation with *S. cerevisiae* + *L. plantarum* (36.27 ARA%, 4.6 times increase) and in the germinated seeds after fermentation with *S. cerevisiae* + *L. plantarum* or *L. plantarum* alone (48.69 and 52.63 ARA%, about 3.7 and 4 times increases, respectively). In amaranth, best results were obtained for seeds after spontaneous fermentation (18.34 ARA%, 5 times increase) and in germinated seeds after fermentation with *S. cerevisiae* + *L. plantarum* or *L. plantarum* alone (46.31 and 52.89 ARA%, 5.1 and 5.8 times increases, respectively). FRAP assays gave similar results in the seeds for the two plants, with significant increase in fermentation with *S. cerevisiae* + *L. plantarum* in quinoa (23.05 mg FSE/g DW, 3.8 times increase) and with *L. plantarum* in amaranth (11.35 mg FSE/g DW, 4.7 times increase). In the germinated seeds, the increase in antioxidant activity was always very high in quinoa in all the fermentation conditions, with the maximum (58.97 mg FSE/g DW, 7 times increase) with *L. plantarum*, while in amaranth germinated seeds, after fermentation with *L. plantarum* and with *S. cerevisiae* + *L. plantarum*, the values were 37.17 and 37.92 mg FSE/g DW, respectively, both about 3 times increases.

The fermentation without sterilization for 48 h (Table 2) confirmed the increase in polyphenols, flavonoids and total antioxidant capacity in all the fermented samples compared to the unfermented ones.

The total content of polyphenols was not further increased by the longer fermentation time compared to 24 h fermentation. The best conditions for quinoa seeds were fermentation with *S. cerevisiae* and *S. cerevisiae* + *L. plantarum*, while for amaranth, maximum values were found for spontaneous fermentation as observed at 24 h. Also, for the germinated seeds, the trend and values were comparable with those obtained at 24 h with the exception of quinoa in which the best condition at 48 h was fermentation with *S. cerevisiae* while at 24 h was spontaneous fermentation.

Total flavonoid content at 48 h increased in respect to fermentation for 24 h, especially in germinated seeds of the two plants, with the highest values in quinoa with spontaneous fermentation (13.33 mg/QE g DW).

Considering total antioxidant activity, DPPH and FRAP assays indicated differences between 24 h and 48 h of fermentation. Although the trend confirmed a general greater antioxidant capacity of the germinated seeds in both plant species, at 48 h, the highest values by DPPH and FRAP in quinoa were found in spontaneous fermentation (70.38 ARA% and 30.53 mg FSE/g DW). In amaranth, the best results were obtained with fermentation with *L. plantarum* (55.3 ARA% and 27 mg FSE/g DW) and with *S. cerevisiae* + *L. plantarum* (43.02 ARA% and 29.87 mg FSE/g DW).

The same determinations were repeated on quinoa and amaranth seeds and germinated seeds fermented for 24 h after sterilization of the plant material to avoid spontaneous fermentation (Table 3). 

Results indicated a general decrease induced by sterilization for the content of polyphenols, flavonoids and antioxidant activity for both species in respect to unsterilized samples. However, germinated seeds showed polyphenol content always significantly higher than seeds for all treatments considered. The content of flavonoids, in both seeds and seedlings of quinoa and amaranth, was always lower than the corresponding content without fermentation. The only exceptions were the high flavonoid values (14.63 mg QE/g DW) in amaranth seeds fermented with *L. plantarum* and in quinoa seeds (12.3 mg QE/g DW) fermented with *S. cerevisiae* + *L. plantarum*. Moreover, results of total antioxidant capacity by DPPH and FRAP assay indicated maximum values in quinoa seeds after fermentation with *S. cerevisiae* + *L. plantarum* (10.96 ARA % and 28.65 mg FSE/g DW) and in amaranth seeds with *L. plantarum* for FRAP assay (20.68 mg FSE/g DW) in accordance with flavonoids. In the germinated seeds of both species, especially the FRAP assay highlighted increased values after 24 h in nearly all fermentation conditions, but in DPPH assay, a general decrease was observed.

Analyses at 48 h of fermentation in sterilized amaranth and quinoa seeds and germinated seeds highlighted the same trend observed at 24 h, with a general decrease in all parameters in respect to the same treatment without sterilization (Table 4). For quinoa seeds, the best treatment resulted from fermentation with *S. cerevisiae* + *L. plantarum* for all parameters analyzed (total polyphenols and flavonoids, DPPH and FRAP), while in amaranth, both fermented seeds and germinated seeds often had the same amount or a decrease in antioxidant molecules and capacity.

### 2.3. Total Content of Mineral Nutrients in Raw, Cooked and Germinated Seeds

**Figure 2:**
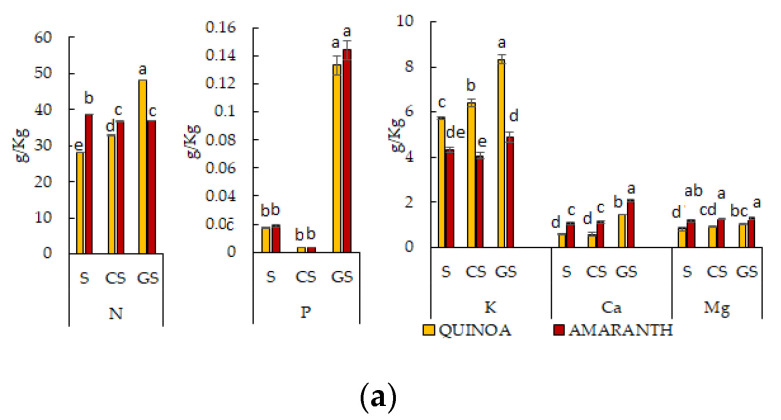

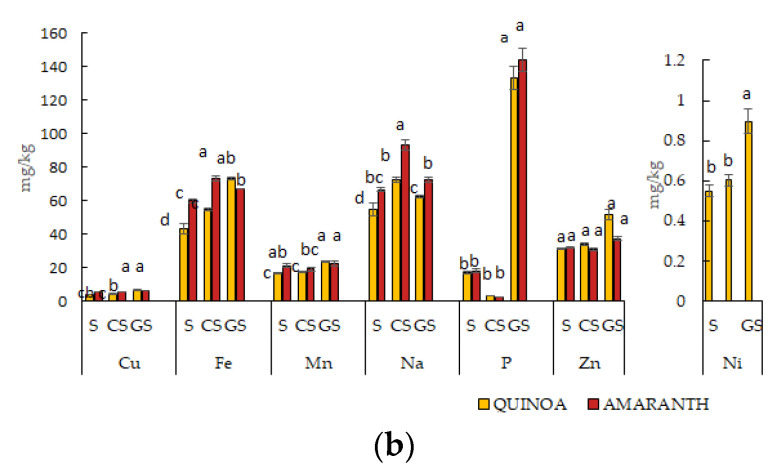
Content of N, P, K, Ca and Mg in quinoa and amaranth seeds (S), cooked seeds (CS) and germinated seeds (GS) (**a**). Content of Cu, Fe, Mn, Na, Zn and Ni (**b**). For the macro- and micronutrients, values expressed as g or mg of element/kg of dry weight of plant sample (g/kg DW or mg/kg DW) were the average of three determinations ± SD. For each element, values followed by different letters were statistically significant according to Tukey’s test (*p* ≤ 0.05).

Macronutrients N, P, K, Ca and Mg (Figure 2a) and micronutrients Cu, Fe, Mn, Na, Zn and Ni (Figure 2b) were determined in raw and cooked seeds and germinated seeds of both species.

Concerning N, germinated seeds of quinoa presented the highest value (about 48 g/kg DW), followed by cooked (about 33 g/kg DW) and raw seeds (about 28 g/kg DW). Instead, amaranth showed the highest values of N in raw seeds (about 39 g/kg DW) and non-significant differences between cooked and germinated seeds (about 36 g/kg DW). Amaranth and quinoa showed higher values of P in the germinated seeds (0.144 g/kg and 0.133 g/kg, respectively) and lower values in raw (about 0.02 g/kg) and in cooked seeds (about 0.0005 g/kg). Germinated seeds of quinoa were the richest in K (8.34 g/kg), while in raw and cooked seeds, the values were 5.74 g/kg and 6.40 g/kg, respectively. In amaranth, K was always significantly lower than in quinoa, with values around 4–5 g/kg. On the contrary, the values of Ca and Mg were significantly higher in amaranth than in quinoa, and the germinated seeds presented the highest values in respect to the raw and the cooked seeds, with 2.07 g/kg and 1.45 g/kg for Ca and 1.2 g/kg and 1.01 g/kg for Mg in amaranth and quinoa, respectively. 

As to the content of micronutrients (Figure 2b), Fe and Na are the most representative in all the samples of both species. Iron had the highest value in the germinated seeds of quinoa and in the cooked seeds of amaranth, with 73.4 mg/kg and 73.5 mg/kg, respectively. Manganese had similar values in all samples of both species, although significantly higher values were found in the germinated seeds of both species (about 23–24 mg/kg), and in the raw and cooked seeds, significantly lower values were found (around 17–19 mg/kg). The highest amount of Zn was detected in the germinated seeds of quinoa (51.7 mg/kg), significantly higher than germinated seeds of amaranth (37.5 mg/kg). Otherwise, no significant differences in Zn content were observed among all the other samples. The germinated seeds of both species evidenced Cu content significantly higher (around 7 mg/kg) than the raw and cooked seeds (around 4–5 mg/kg in quinoa and of 6 mg/kg in amaranth). Concerning Na, amaranth always showed significantly higher values than quinoa. The highest value was observed in cooked seeds of amaranth (93.2 mg/kg) and the least in raw seeds of quinoa (54.7 mg/kg). Lastly, it is interesting to point out that Ni was not detected by the ICP-OES technique in amaranth but was observed in trace in quinoa samples, with values in the germinated seeds significantly higher than in raw and cooked ones (0.90, 0.60 and 0.55 mg/kg, respectively).

### 2.4. Mutagenicity and Antimutagenicity Assays with D7 Strain of the Yeast S. cerevisiae 

**Figure 3:**
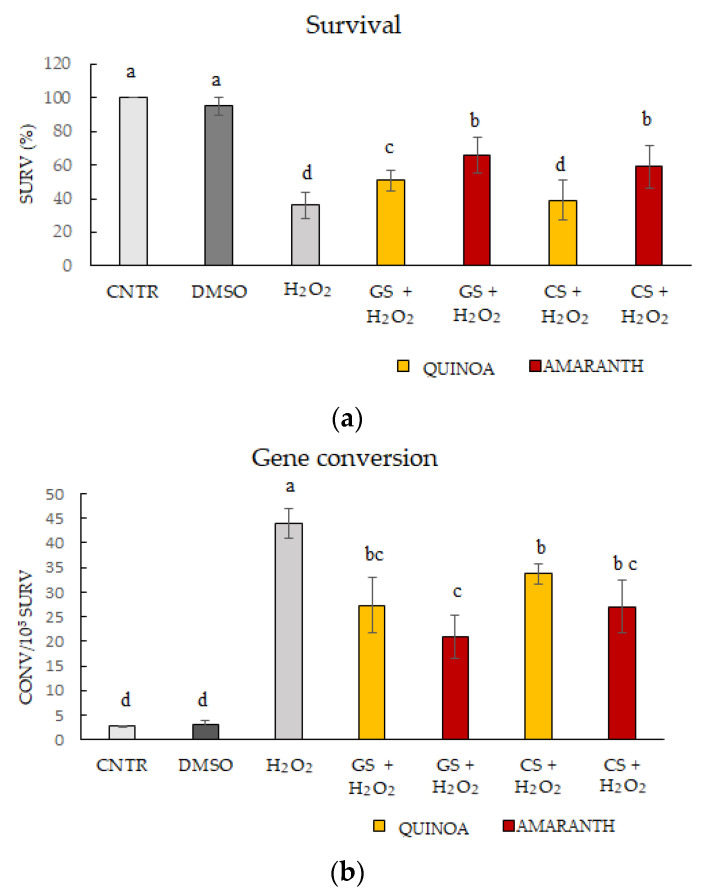

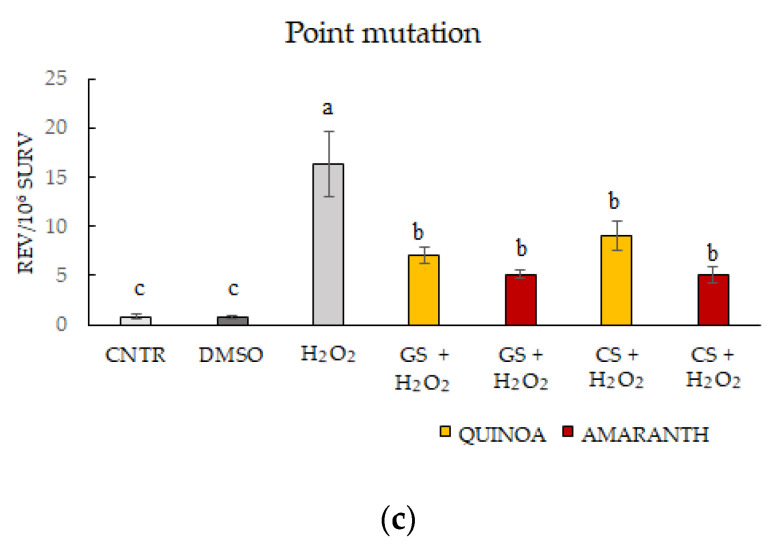
Survival percentage in D7 strain of *Saccharomyces cerevisiae* (**a**). Frequency of gene conversion (convertants/10^5^ survivals) (**b**). Frequency of point mutation (revertants/10^6^ survivals) (**c**) in control (CNTR, white column), negative control with DMSO solvent (DMSO, dark grey column), treatments with 4 mM hydrogen peroxide (H_2_O_2,_ grey column) and treatments with 1 mg/mL quinoa and amaranth extracts from germinated seeds (GS) and cooked seeds (CS) in presence of H_2_O_2_. Values were expressed as the mean ± SD of three independent experiments. Values followed by different letters were statistically significant with Tukey’s test for *p* ≤ 0.05.

Neither toxicity nor increases in the gene conversion and point mutation frequency were found in the control with DMSO solvent (DMSO). Quinoa and amaranth germinated and cooked seeds did not determine toxic effect in all the experimental conditions. Compared to DMSO solvent, no significant survival decrease and mutagenicity were observed in the presence of the extracts at the two concentrations examined (1 mg/mL, 2 mg/mL). Since the samples were neither toxic nor mutagens, antimutagenesis tests were set up with a single dose (1 mg/mL) of quinoa and amaranth extracts and with oxidant agent H_2_O_2_ (4 mM). As reported in Figure 3a, the oxidizing agent caused a significant decrease in survival, about 70%. By adding the plant extracts of germinated seeds of both species and cooked seeds of amaranth, survival percentage significantly increased. 

In Figure 3b, gene conversion results are shown. The values of convertants ranged from 2.69 convertants/10^5^ survivals of the control to 43.93 convertants/10^5^ survivals of the oxidant agent, with an increase of 19 times. The addition of H_2_O_2_ in yeast culture with the quinoa and amaranth extracts determined a significant decrease in the gene conversion frequency compared to the oxidizing agent only. The amaranth germinated seed extract decreased by 50% the number of convertants compared to the oxidizing agent. Quinoa GS and amaranth CS also caused a decrease in the gene conversion frequency, with values of 27.34% and 20.93%, respectively. Compared to the oxidizing agent, however, quinoa CS (33.73 convertants/10^5^ survivals) had a less marked effect in decreasing the frequency of gene conversion. This phenomenon can be related to the lower survival rate.

Results of point mutation frequencies are reported in Figure 3c. H_2_O_2_ oxidizing agent led to an increase in revertants of about 16 times higher in respect to the control. All plant extracts caused a significant decrease in the frequency of point mutation compared to the oxidizing agent. No significant statistical differences were observed between the different extracts.

## 3. Discussion

The results obtained from the determinations of antioxidant molecules and antioxidant activity in quinoa and amaranth seeds, cooked seeds and germinated seeds were in line with previous studies indicating that quinoa seeds presented higher values than amaranth seeds and that sprouts were the samples with the highest polyphenol content [20,36,41]. Moreover, in seeds and germinated seeds of quinoa, the flavonoid content was higher than in amaranth samples, as already reported [32]. Our results confirmed that the germination process naturally increased antioxidant compounds capable of providing health benefits [34,35,36]. However, a lower flavonoid content in var. Tunkahuan seeds of quinoa (9.48 mg QE/100 g FW) than that obtained in our study (about 3 mg QE/ g FW) was formerly detected [42], while the content of total polyphenols in their studies was about seven times higher. It should be considered that in other studies, much variability in the amount of total flavonoid content in the seeds of different varieties of quinoa and amaranth was observed [22]. 

From our results, the antioxidant activity evaluated by DPPH and FRAP of both quinoa and amaranth germinated seeds was greater than that of the seeds, in line with previous studies [17,32,36,43]. No significative effect of cooking was observed for the content in antioxidant molecules and antioxidant activity. 

Previous HPLC analysis of seeds and sprouts of quinoa and amaranth [32] determined the main phenolic acid present in both species, like gallic acid, p-Hydroxybenzoic acid, vanillic acid, p-coumaric acid, caffeic acid and cinnamic acid (in the seeds) and p-coumaric acid, syringic acid and ferulic acid (in the sprouts). The main flavonoids determined in quinoa and amaranth sprouts were rutin, vitexin, isovitexin and morin, while in the seeds of both species, orientin, vitexin, isovitexin, morin and traces of hesperidin and neohesperidin were detected [32]. The presence of the abovementioned molecules could potentially be responsible for the antioxidant activity detected.

Among macronutrients, significant higher values of K (1.5 times increase) and N (1.7 times increase) were observed in the germinated seeds of quinoa and higher values of P, Ca and Mg (8, 1.9 and 1.2 times increases, respectively) were observed in the germinated seeds of amaranth, in respect to the corresponding raw and cooked seeds. This increase could be explained by the mobilization of cotyledonary seed reserves which occurs during seed germination [44]. The macro- and micronutrients of quinoa var. Tunkahuan were recently analyzed [42], considering leaves and seeds where statistically significant differences in mineral content were detected and quinoa leaves that had a higher content of Ca, K, Fe and Zn than the seeds. The abovementioned results therefore demonstrated that the mineral content depends on the varieties, the physiological stage, the plant tissues and the cooking methods considered [29,42,45]. 

In our results, Ni was present in traces in the quinoa Tunkahuan but not detected in amaranth Alegria, while in previous work on different varieties, a comparable amount of Ni (about 0.16 mg /kg) was found in the seeds of the two plants [46]. Low levels or Ni absence can be very interesting with regards to human nutrition and Ni sensitivity or toxicity [47].

Concerning fermentation experiments for 24 or 48 h with the yeast *S. cerevisiae*, with the bacterium *L. plantarum* and with their combination, our results indicated that fermentation was an effective method for increasing the total polyphenols, in accordance with previous observations [48,49]. Recently, experiments on quinoa fermentations based on *Saccharomyces* spp. and *Lactobacillus* spp. highlighted how both the time and the type of inoculation were decisive for the content of bioactive molecules in fermented products and that short times of fermentation were more efficient than prolonged ones [50]. This demonstrates that the fermentation process positively influences the antioxidant capacity both through the increase in phenolic and/or flavonoid compounds (induced by the microbial hydrolysis reaction) and through their synthesis by microorganisms [34,39]. In a previous work [51], fermentation in quinoa for 24 to 48 h recorded a decrease in polyphenol content, while in amaranth, flavonoids were not even detected. On the contrary, other studies [52] reported that the amaranth samples fermented for 48 h were better than those fermented for 24 h, demonstrating that the antioxidant capacity varied depending on the experimental conditions and the plant/variety examined. In our experiments, 24 h fermentation had the greatest impact on the increase in total antioxidant capacity. Specifically, the maximum values were found in sprouts fermented by *L. plantarum* bacterium, followed by the ones fermented by *L. plantarum* + *S. cerevisiae*. The values dropped after 48 h, and the differences between all fermentation conditions (spontaneous, with only bacterium, with only yeast and with yeast + bacterium) were less evident. When the raw and germinated seeds were sterilized before fermentation, excluding spontaneous fermentation, a general lowering in the total antioxidant capacity was noticed both at 24 h and 48 h. The elimination of microorganisms naturally present in seeds and sprouts had evident effects on antioxidant molecule content, with the highest content found in fermented seeds. On the contrary, in wheat germ, time was a determining factor together with pH: using either *S. cerevisiae* or *L. plantarum* as fermentative microorganism, the anti-radical activity (expressed as %) increased from 24 to 48 h [53]. In fact, by operating at a pH around neutrality, in many samples, the ARA value increased by approximately 20% (for example, with *L. plantarum* from 61.08% to 78.13%) [53]. Moreover, in amaranth samples, it has been shown that the antioxidant capacity varied depending on the experimental conditions and particularly on the fermentation time: the samples fermented for 48 h had higher capacity than those fermented for 24 h [52]. Other reports [54] confirmed the importance of fermentation time in quinoa since in all the varieties analyzed, the time factor determined an increase in antioxidant activity measured by the DPPH assay.

Quinoa and amaranth extracts (germinated and cooked seeds) were not mutagenic and were effective in reducing the damage caused by H_2_O_2_, both against gene conversion and point mutation, showing protective effects except in the cooked seeds of quinoa. It could therefore be assumed that the bioactive compounds present in the extracts of both plant species played a protective role against oxidative damage. The results obtained were in line with data previously reported on vegetable matrices [55,56,57]. 

## 4. Materials and Methods

### 4.1. Plant Material 

Seeds were obtained from the Instituto Nacional de Investigaciones Agropecuaria (INIAP), Ecuador, and maintained in the INIAP germplasm bank. The investigated varieties were *Chenopodium quinoa* Willd., Tunkahuan variety (germplasm bank code ECU 0621), and *Amaranthus caudatus* L., Alegrìa variety (germplasm bank code ECU 2210). 

### 4.2. Seed Germination and Cooking Procedure

Seeds were germinated in Petri dishes containing two sheets of Whatman filter papers moistened with 2 mL of distilled water and kept in the dark at 37 °C for 2 days. Seeds were also cooked in distilled water for 15–20 min, considering the traditional cooking time. Both cooked seeds and germinated seeds were drained with absorbent paper to eliminate the excess of water and weighed to determine fresh weight (FW). Samples were then oven-dried at 42 °C until constant weight, then dry weight (DW) was determined.

### 4.3. Fermentation and Sterilization Procedure

Both cooked and germinated seed samples were fermented in water in four different ways: (1) natural fermentation for considering the activity of native microorganisms, (2) with the yeast *Saccharomyces cerevisiae*, (3) with the bacterium *Lactobacillus plantarum* and (4) with both *S. cerevisiae* and *L. plantarum*. 

All dry plant samples (1 g) were ground using a mortar and placed in distilled water (20 mL) for fermentation without inoculation or with the two microorganisms added in appropriate concentrations: 10^6^ cells/mL solution for *S. cerevisiae*, obtained from commercial yeast, and 10^8^ cells/mL of solution for *L. plantarum* strain MB91 (Scardovi Collection of Bifidobacteria available at the Institute of Agricultural and Technical Microbiology, Department of Agriculture and Food Science of the University of Bologna), kindly provided by Dr. Luisa Pozzo (IBBA-CNR Pisa), grown for 24 h on MRS medium at 37 °C in anaerobic conditions. All samples were fermented following protocols with appropriate modifications [34,58]. Samples were kept under agitation at 30 °C for 24 and 48 h and then dried at 50 °C until constant weight. The flours from ground dry seeds and germinated seeds (1 g in 20 mL of distilled water) were also sterilized in autoclave for 15′ at 0.5 atm before fermentation.

### 4.4. Preparation of Extracts

For antioxidant activity determination, quinoa and amaranth samples were homogenized with an Ultra Turrax homogenizer (Kinematica Polytron PT MR 2100, Lucerne, Switzerland) in 10 mL of 80% EtOH to a final concentration of 1:10 for not fermented samples and 0.25:10 for fermented ones. The samples were shaken overnight and centrifuged at 2000 rcf for half an hour at 4 °C. The recovered supernatant was stored at 4 °C until used for spectrophotometric determinations. For tests with *S. cerevisiae*, supernatant was dried with a rotary evaporator (Labconco, Kansas City, MO, USA) and resuspended in DMSO (dimethyl sulfoxide).

All the following determinations were carried out in three replicates using a UV/visible spectrophotometer (Perkin Elmer, Victor TM X3 apparatus, Waltham, MA, USA).

### 4.5. Total Polyphenol Content

The colorimetric quantification of total polyphenols was determined by the assay of Singleton and Rossi [59]. In particular, 33 μL of extract of each sample was added to 1 mL Folin-Ciocalteau (Merck, Sigma-Aldrich, GmbH, Sternheim, Germany) (diluted 1:5 with distilled water). After 6′ of incubation in the dark at room temperature, 660 μL of 20% sodium carbonate (Na_2_CO_3_) was added and incubated for 1 h in the dark at room temperature. The absorbance was measured at 760 nm against a blank of 80% EtOH. The result was expressed in mg of gallic acid equivalents (mg GaE/g DW) of extract, through the calibration curve of gallic acid.

### 4.6. Total Flavonoid Content

Total flavonoids were measured according to the colorimetric method of Heimler et al. [60]. First, 150 μL of extract of each sample was added to 600 μL of distilled water and 45 μL of 5% nitric sodium (NaNO_2_) in distilled water. After 5 min, 45 μL of 10% aluminum chloride (AlCl_3_) was added. After 6 min, 300 μL of 1M sodium hydroxide (NaOH) in distilled water and 360 μL of distilled water were added. After a final incubation for 30 min, the absorbance was measured at 510 nm against a blank of 80% EtOH. Total flavonoid content was expressed as milligram quercetin equivalents (mg QE/g FW or DW) of extract, through the calibration curve of quercetin.

### 4.7. DPPH Assay

Antioxidant activity was determined by the method of Boudjou et al. [61]. Thirty μL of each extract was added to 1470 μL of DPPH (2.2-Diphenyl-1-picrylhydrazyl) (Merck, Sigma-Aldrich, GmbH, Sternheim, Germany). After incubation in the dark at room temperature for 60 min, absorbance was measured at 517 nm against a blank of 80% EtOH. The anti-radical activity (ARA) was expressed as a percentage of inhibition of the DPPH* radical determined through the following equation:

ARA = 100 ∗ [1 − (Abs of sample/Abs of control)]

### 4.8. FRAP Assay

For FRAP (Ferric Reducing Antioxidant Power) assay, according to the Benzie and Strain protocol [62], 50 μL of extract of each sample was added to 1.5 mL of FRAP, a solution of iron chloride (FeCl_3_), ferril tripyridyltriazine (FeIII-TPTZ) (Merck, Sigma-Aldrich, GmbH, Sternheim, Germany) and acetate buffer, formed by 300 mM of sodium acetate trihydrate (CH_3_COONa × 3 H_2_O) in distilled water at pH 3.6 and 10 mM of glacial acetic acid (CH_3_COOH) in distilled water. After incubation in the dark for 30 min, the absorbance was measured at 593 nm against a blank of 80% EtOH. Results were expressed in milligrams of iron equivalents per gram of dry weight of sample (mg Fe/g DW) of extract, through the calibration curve with ferrous sulphate (FeSO_4_ × 7 H_2_O) standard.

### 4.9. Mineral Nutrient Content 

Raw, cooked and germinated seeds of both quinoa and amaranth were washed in deionized water, oven-dried until constant weight and weighed for the biomass determination. The dried samples were powdered (<1 mm). A digestion method was performed following the protocol previously described [63], with overnight pre-digestion in a mixture of HNO_3_/H_2_O_2_ (2.5:1, *v*/*v*) and microwave-assisted acidic digestion using a microwave Ethos 900 (FKV Srl, Bergamo, Italy). Once the acid was digested, samples were analyzed for the mineral nutrient content using an Inductively Coupled Plasma Emission Spectroscopy (ICP-OES, 5900 Agilent, Santa Clara, CA, USA), and the final value is reported in grams or milligrams of mineralized material (g or mg/kg). 

### 4.10. Yeast Test 

The diploid D7 strain [(a: ade 2–40 trp 5–12 ilv 1–92)/(α: ade 2–119 trp 5–27 ilv 1–92)] of the yeast *S. cerevisiae*, obtained from Zimmermann et al. [64], was employed in the genotoxicity (toxicity and mutagenesis) and antimutagenic assays (protective effects) [65,66]. Mitotic gene conversion (GC) and point reverse mutation (PM) were measured at the *trp-5* and at the *ilv1–92 loci*, respectively. GC and PM were detected by the appearance on selective media of colonies not requiring tryptophan or isoleucine. Protective and antimutagenic effects of cooked seeds and shoots of both varieties’ extracts were evaluated against H_2_O_2_-induced mutagenesis. Stock cultures were obtained by inoculating about 107 cells ml^−1^ in liquid complete medium (1% glucose, 2% bactopeptone, 0,5% yeast extract) and incubating at 30 °C overnight up to the logarithmic phase or for 48 h up to the stationary phase. The composition of the selective synthetic medium plates was 0.6% Bacto yeast nitrogen base (without amino acids) (Difco, Becton Dickinson, Milan, Italy), 1% glucose, 0.12% adenine and 0,25% isoleucine (on the selective medium for gene conversion) or 0,4% tryptophan (on the selective medium for point mutation). Aliquots of these cultures were counted and plated, after suitable dilutions, on complete and selective media to determine survival, *trp convertants* and *ilv revertants*. Antimutagenesis assay in yeast cells was performed by the same procedure as the mutagenesis test reported. After the reaching logarithmic phase, H_2_O_2_ (4 mM) was added to the flasks and stirred for 90 min at 30 °C in the presence of extracts. Samples examined were control (only yeast cells), negative control with DMSO (extracts solvent), with H_2_O_2_, in presence of extracts of cooked quinoa and amaranth seeds (1 mg/mL) with and without H_2_O_2_, and in presence of quinoa and amaranth sprouts (1 mg/mL) with and without H_2_O_2_. Cell counting was carried out under an optical microscope and subsequently was diluted, then plating was carried out on the three media.

### 4.11. Statistical Analysis

For each experiment, three replications were performed and results reported as the mean of three determinations ± SD (standard deviation). Analysis of variance and Tukey’s post hoc test for multiple comparisons were used to identify statistically significant differences between treatments and between the two species, using version 6.0 of the Statistica package (StatSoft, Hamburg, Germany). Different letters indicate significant differences at *p* ≤ 0.05. 

## 5. Conclusions

In this work, the nutraceutical value of *Chenopodium quinoa* var. Tunkahuan and *Amaranthus caudatus* var. Alegrìa, native plant species cultivated in Ecuador, was evaluated. Seeds and germinated seeds of both plant species, raw or after cooking, fermentation and sterilization, were analyzed both in terms of bioactive molecules (polyphenols and flavonoids) and total antioxidant capacity via DPPH and FRAP assays.

The results demonstrated that germination induced an increase in bioactive compounds and antioxidant properties in the two plants, as did fermentation, which determined a notable increase depending on both the type of fermentative microorganism used and the plant substrate (seeds or germinated seeds). In general, we can state that quinoa showed higher levels of polyphenols, flavonoids and antioxidant activity than amaranth, especially in the sprouts fermented spontaneously for 24 h. The prolonged time of fermentation was not decisive for the increase in the aforementioned compounds; on the contrary, it was noted that after 48 h, in many cases, these tended to decrease. Sterilization before fermentation resulted, however, in a clear decrease in bioactive compounds compared to non-sterilized samples but higher amounts when compared to the same non-fermented samples. The results obtained therefore suggest that fermentation increases the antioxidant content and consequently the potential beneficial effect of fermented products based on quinoa and amaranth.

The analysis of the macronutrients highlighted that the K content was significantly higher in quinoa than in amaranth, while in the latter, higher levels of Ca and Mg were determined. Nitrogen, on the other hand, obtained the highest values in the raw seed and cooked seed of amaranth and in the germinated seeds of quinoa. Regarding micronutrients, the two plants have a similar composition except for the absence of Ni in amaranth. This feature is noteworthy, as Ni is an element that can induce intolerances or allergies. We can therefore conclude that the mineral content depends on the variety, the physiological stage, the plant tissues and the cooking methods considered.

Neither toxic nor mutagenic effects were detected in the tests with *S. cerevisiae* D7 strain for extracts from cooked and germinated seeds of quinoa and amaranth, which were also found to be antimutagenic, in accordance with the presence of antioxidant molecules and anti-radical action determined in the two plants.

In vitro studies were useful to characterize the two plants in relation to the possible beneficial effects of the analyzed samples, but further investigations on cell cultures and in vivo systems would be necessary to confirm positive effects on human health.

## Figures and Tables

**Table 1:** Fermentation results at 24 h without sterilization. Content of total polyphenols (mg GaE/g DW), total flavonoids (mg QE/g DW), DPPH (ARA%) and FRAP (mg FSE/g DW) determined on seeds (S) and germinated seeds (GS) (non-fermented NF) of quinoa and amaranth and after spontaneous fermentation (Sp), in samples inoculated with *Saccharomyces cerevisiae* (Sc), with *Lactobacillus plantarum* (Lp) and with both fermenting microorganisms (Sc + Lp). Values were the average of three determinations ± SD; values followed by different letters within columns were statistically significant according to Tukey’s test (*p* ≤ 0.05).

Fermentation Results at 24 h (No Sterilization)								
	Total Polyphenols (mg GaE/g DW)		Total Flavonoids (mg QE/g DW)		DPPH (ARA%)		FRAP (mg FSE/g DW)	
	Quinoa	Amaranth	Quinoa	Amaranth	Quinoa	Amaranth	Quinoa	Amaranth
S NF	1.55 ± 0.03 i	0.54 ± 0.06 j	3.00 ± 0.14 kl	2.35 ± 0.33 lm	7.89 ± 0.45 h	3.7 ± 1.18 i	6.07 ± 0.04 i	2.4 ± 0.13 j
S Sp	7.49 ± 0.14 de	5.59 ± 0.21 f	4.43 ± 0.11 ghi	6.1 ± 0.05 cd	19.05 ± 0.73 f	18.34 ± 0.1 f	6.19 ± 0.24 i	8.96 ± 0.54 g
S Sc	6.82 ± 0.96 e	4.55 ± 0.19 g	4.22 ± 0.14 hi	1.58 ± 0.22 mn	17.76 ± 0.33 f	13.18 ± 0.94 g	3.90 ± 0.13 j	4.02 ± 0.28 j
S Lp	4.39 ± 0.44 g	5.12 ± 0.48 fg	1.68 ± 0.08 mn	3.26 ± 0.15 jk	7.51 ± 0.16 hi	15.72 ± 0.67 fg	7.00 ± 0.4 hi	11.35 ± 0.39 f
S Sc + Lp	10.54 ± 0.41 b	5.22 ± 0.22 fg	4.87 ± 0.27 efgh	1.31 ± 0.09 n	36.27 ± 2.05 d	7.82 ± 0.62 h	23.05 ± 0.54 d	6.48 ± 0.7 hi
GS NF	2.60 ± 0.04 h	2.54 ± 0.16 h	4.46 ± 0.43 fghi	4.9 ± 0.173 efgh	13.07 ± 1.41 g	9.06 ± 1.25 h	8.31 ± 0.24 gh	12.7 ± 0.2 f
GS Sp	11.78 ± 0.42 a	9.92 ± 0.33 bc	12.81 ± 0.31 a	9.41 ± 0.49 b	38.79 ± 1.28 d	27.46 ± 2.17 e	23.76 ± 0.315 d	16.16 ± 0.83 e
GS Sc	8.07 ± 0.68 d	4.62 ± 0.17 fg	4.06 ± 0.14 hij	3.59 ± 0.44 ijk	24.33 ± 0.78 e	15.45 ± 0.89 fg	24.96 ± 0.37 d	6.8 ± 0.09 hi
GS Lp	9.70 ± 0.21 bc	9.44 ± 0.04 c	5.14 ± 0.04 efg	5.35 ± 0.07 def	52.63 ± 1.84 ab	52.89 ± 2.76 a	58.97 ± 1.13 a	37.17 ± 1.22 c
GS Sc + Lp	8.35 ± 0.68 d	6.64 ± 0.27 e	6.65 ± 0.42 c	5.46 ± 0.02 de	48.69 ± 0.91 bc	46.31 ± 0.91 c	49.83 ± 0.03 b	37.92 ± 1.17 c

**Table 2:** Fermentation results at 48 h without sterilization. Content of total polyphenols (mg GaE/g DW), total flavonoids (mg QE/g DW), DPPH (ARA%) and FRAP (mg FSE/g DW) determined on seeds (S) and germinated seeds (GS) (non-fermented NF) of quinoa and amaranth and after spontaneous fermentation (Sp), in samples inoculated with *Saccharomyces cerevisiae* (Sc), with *Lactobacillus plantarum* (Lp) and with both fermenting microorganisms (Sc + Lp). Values were the average of three determinations ± SD; values followed by different letters within columns were statistically significant according to Tukey’s test (*p* ≤ 0.05).

Fermentation Results at 48 h (No Sterilization)								
	Total Polyphenols (mg GaE/g DW)		Total Flavonoids (mg QE/g DW)		DPPH (ARA%)		FRAP (mg FSE/g DW)	
	Quinoa	Amaranth	Quinoa	Amaranth	Quinoa	Amaranth	Quinoa	Amaranth
S NF	1.55 ± 0.03 ij	0.54 ± 0.06 j	3.00 ± 0.14 hi	2.35 ± 0.33 i	7.89 ± 0.45 hij	3.7 ± 1.18 j	6.07 ± 0.04 kl	2.4 ± 0.13 m
S Sp	2.15 ± 0.14 i	4.63 ± 0.07 f	4.23 ± 0.03 fgh	2.04 ± 0.07 i	14.25 ± 0.88 gh	19.63 ± 0.92 g	5.27 ± 0.08 l	17.38 ± 0.78 g
S Sc	6.86 ± 0.77 de	3.26 ± 0.33 gh	6.50 ± 0.24 de	6.2 ± 0.29 e	27.35 ± 1.09 f	17.68 ± 1.29 g	18.06 ± 0.37 g	12.7 ± 0.33 hi
S Lp	4.31 ± 0.35 fg	2.6 ± 0.17 hi	3.51 ± 1.49 ghi	4.23 ± 0.17 fgh	6.95 ± 1.06 ij	15.94 ± 1.69 g	10.52 ± 0.64 ij	8.99 ± 0.29 j
S Sc + Lp	6.70 ± 0.03 de	2.29 ± 0.2 hi	3.37 ± 0.14 ghi	5.79 ± 0.23 ef	37.34 ± 2.35 e	7.39 ± 1.79 ij	13.41 ± 0.79 h	8.8 ± 0.6 jk
GS NF	2.60 ± 0.04 hi	2.54 ± 0.16 hi	4.46 ± 0.43 fgh	4.9 ± 0.173 efg	13.07 ± 1.41 ghi	9.06 ± 1.25 hij	8.31 ± 0.24 jk	12.71 ± 0.2 hi
GS Sp	9.69 ± 0.59 c	9.28 ± 0.2 c	13.33 ± 0.17 a	10.52 ± 0.35 bc	70.38 ± 2.79 a	29.02 ± 2.05 f	30.53 ± 0.18 ab	21.4 ± 0.78 f
GS Sc	12.87 ± 0.34 a	7.38 ± 0.19 c	10.29 ± 0.61 bc	9.9 ± 0.46 c	47.42 ± 0.81 cd	38.7 ± 0.57 e	27.33 ± 0.94 cde	24.66 ± 0.44 e
GS Lp	11.04 ± 0.64 b	5.76 ± 0.15 e	10.79 ± 0.18 bc	10.56 ± 0.56 bc	52.02 ± 2.19 bc	55.3 ± 2.03 b	31.57 ± 0.8 a	27 ± 1.6 de
GS Sc + Lp	11.02 ± 0.66 b	7.23 ± 0.13 d	8.13 ± 0.84 d	11.61 ± 0.6 b	46.39 ± 1.37 cd	43.02 ± 8.62 de	28.17 ± 2.25 bcd	29.87 ± 0.8 abc

**Table 3:** Fermentation results at 24 h, after sterilization. Content of total polyphenols (mg GaE/g DW), total flavonoids (mg QE/g DW), DPPH (ARA%) and FRAP (mg FSE/g DW) determined on seeds (S) and germinated seeds (GS) (non-fermented NF) of quinoa and amaranth, and after inoculation with *Saccharomyces cerevisiae* (Sc), with *Lactobacillus plantarum* (Lp) and with both fermenting microorganisms (Sc + Lp). Values were the average of three determinations ± SD; values followed by different letters within columns were statistically significant according to Tukey’s test (*p* ≤ 0.05).

Fermentation Results at 24 h (After Sterilization)								
	Total Polyphenols (mg GaE/g DW)		Total Flavonoids (mg QE/g DW)		DPPH (ARA%)		FRAP (mg FSE/g DW)	
	Quinoa	Amaranth	Quinoa	Amaranth	Quinoa	Amaranth	Quinoa	Amaranth
S NF	1.55 ± 0.03 f	0.54 ± 0.06 h	3 ± 0.14 de	2.35 ± 0.33 e	7.89 ± 0.45 bc	3.7 ± 1.18 fg	6.07 ± 0.04 f	2.4 ± 0.13 g
S Sc	2.79 ± 0.05 e	2.65 ± 0.13 e	1.99 ± 0.03 ef	1.64 ± 0.37 ef	5.54 ± 0.1 cdef	1.09 ± 0.96 g	4.98 ± 0.17 f	2.54 ± 0.18 g
S Lp	2.49 ± 0.16 e	0.92 ± 0.06 gh	1.85 ± 0.42 ef	14.63 ± 1.65 a	4.3 ± 0.15 defg	1.56 ± 1.4 fg	5.07 ± 0.13 f	20.68 ± 1.25 b
S Sc + Lp	5.77 ± 0.30 b	1.22 ± 0.04 fg	12.3 ± 0.48 b	0.69 ± 0.12 f	10.96 ± 2.5 ab	0.41 ± 0.39 g	28.65 ± 0.65 a	2.42 ± 0.13 g
GS NF	2.60 ± 0.04 e	2.54 ± 0.16 e	4.46 ± 0.43 cd	4.9 ± 0.173 c	13.07 ± 1.41 a	9.1 ± 1.25 abc	8.31 ± 0.24 e	12.71 ± 0.2 d
GS Sc	7.38 ± 0.04 a	4.45 ± 0.12 cd	1.98 ± 0.13 ef	2.58 ± 0.09 e	7.59 ± 2.6 bcde	4.1 ± 1.4 defg	14.09 ± 0.29 cd	9.56 ± 0.47 e
GS Lp	4.98 ± 0.15 c	4.11 ± 0.04 d	2.71 ± 0.05 e	2.27 ± 0.3 ef	7.8 ± 0.33 bcde	7.3 ± 1.1 bcde	14.87 ± 0.5 c	19.71 ± 0.45 b
GS Sc + Lp	4.69 ± 0.11 cd	5.21 ± 0.22 cd	2.42 ± 0.15 e	2.13 ± 0.17 ef	4.31 ± 2.7 defg	5.5 ± 1.3 cdef	10.2 ± 1.46 e	15.61 ± 0.3 c

**Table 4:** Fermentation results at 48 h, after sterilization. Content of total polyphenols (mg GaE/g DW), total flavonoids (mg QE/g DW), DPPH (ARA %) and FRAP (mg FSE/g DW) determined on seeds (S) and germinated seeds (GS) (non-fermented NF) of quinoa and amaranth, and fermentation of samples inoculated with *Saccharomyces cerevisiae* (Sc), with *Lactobacillus plantarum* (Lp) and with both fermenting microorganisms (Sc + Lp). Values were the average of three determinations ± SD; values followed by different letters within columns were statistically significant according to Tukey’s test (*p* ≤ 0.05).

Fermentation Results at 48 h (After Sterilization)								
	Total Polyphenols (mg GaE/g DW)		Total Flavonoids (mg QE/g DW)		DPPH (ARA%)		FRAP (mg FSE/g DW)	
	Quinoa	Amaranth	Quinoa	Amaranth	Quinoa	Amaranth	Quinoa	Amaranth
S NF	1.55 ± 0.03 g	0.54 ± 0.06 h	3 ± 0.14 d	2.35 ± 0.33 de	7.89 ± 0.45 bc	3.7 ± 1.18 de	6.07 ± 0.04 ef	2.4 ± 0.13 gh
S Sc	3.0 ± 0.14 ef	4.33 ± 0.43 cd	1.46 ± 0.05 ef	0.55 ± 0.29 f	2.49 ± 1.1 def	0.85 ± 0.07 ef	4.98 ± 0.12 f	2 ± 0.2 h
S Lp	2.5 ± 0.17 f	1.07 ± 0.14 gh	1.98 ± 0.13 de	8.55 ± 0.49 a	2.2 ± 0.87 def	1.47 ± 0.1 ef	4.73 ± 0.11 fg	11.09 ± 0.87 d
S Sc + Lp	6.24 ± 0.26 a	1.5 ± 0.1 g	8 ± 0.77 a	0.41 ± 0.15 f	15.9 ± 0.63 a	0.42 ± 0.45 f	23.46 ± 1.8 a	2.34 ± 0.03 h
GS NF	2.6 ± 0.04 f	2.54 ± 0.16 f	4.46 ± 0.43 bc	4.91 ± 0.17 b	13.07 ± 1.41 a	9.06 ± 1.25 b	8.31 ± 0.24 e	12.71 ± 0.2 d
GS Sc	4.81 ± 0.2 bc	5.03 ± 0.22 b	2 ± 0.38 de	2.78 ± 0.07 de	4.95 ± 2.59 cd	3.16 ± 1.12 def	13.18 ± 0.32 cd	12.9 ± 0.51 cd
GS Lp	4.81 ± 0.22 bc	2.74 ± 0.17 ef	2.27 ± 0.06 de	5.58 ± 0.54 b	0.82 ± 0.83 ef	8.45 ± 1.1 b	17 ± 0.68 b	16.7 ± 1.14 b
GS Sc + Lp	3.17 ± 0.14 e	4.12 ± 0.22 d	3.12 ± 0.95 cd	3.21 ± 0.34 cd	0.23 ± 0.39 f	0.73 ± 0.43 ef	15.2 ± 0.86 bc	16.72 ± 0.67 b

## Data Availability

The original contributions presented in the study are included in the article; further inquiries can be directed to the corresponding author.
